# New burns and trauma journal celebrating translational research

**DOI:** 10.4103/2321-3868.118922

**Published:** 2013-09-18

**Authors:** Antonino Gullo, Giovanni Li Volti, Giuseppe Ristagno

**Affiliations:** 1 Departments of Anesthesia and Intensive Care, Catania School of Medicine, USA; 2 Drug of Sciences, Section of Biochemistry, University of Catania, Catania, USA; 3 Cardiovascular Research, Mario Negri Institute, Milan, Italy; 4 Rancho Mirage, Weil Institute of Critical Care Medicine, LA, CA, USA; 5 Department of Anesthesia and Intensive Care, Catania School of Medicine, University-Hospital, Csatania, 95126 78, S. Sofia Street Building No.1, Italy

**Keywords:** Burns, epistemology, ontology, science communication, trauma, translational research, translational medicine

## Abstract

Welcome to the journal of Burns & Trauma launched in 2013 and published by the prestigious Wolters Kluwer Health. We are grateful to promote a cultural challenge toward a new horizon in the field of translational research (TR). We enjoy to work together with the common objective to perform continuous medical education programs, exploring the methods in research, designing study, and to improve multidisciplinary and multiprofessional collaboration in the basic sciences and in the clinical trials. Defned narrowly, epistemology is the study of knowledge and justified belief. Epistemology is concerned with the following questions: What are the necessary and sufcient conditions of knowledge? What are its sources? What is its structure and what are its limits? More broadly, epistemology is about issues having to do with the creation and dissemination of knowledge in particular areas of inquiry as defned and revisiting at the beginning of the last decades. Translational medicine (TM) should meet the demands to maintain or expanding the biomedical workforce and education programs that attract and retain young people in the translational and biomedical sciences. With this present contributes, we invite the members of the editorial board of Burns & Trauma to encourage submitting, in a special section, their personal experience about the philosophy of “Translation research.” If this has a chance, welcome to the researchers, clinicians, and the allied people for their decisive contributions to strengthen the importance of a common way about the principles and methods of basic and clinical research. TR and TM represent a dynamic entity making a link, a sort of bridge, “from bench to bedside”, or from laboratory experiments through clinical trials to point-of-care patient applications. Epistemological pluralism is a critical point for conducting innovative, collaborative research which can lead to more successful integrated and successfully study, particularly important in the feld of burns and trauma.

## Introduction

Welcome to the journal of Burns & Trauma launched in June 2013 and published by the prestigious Wolters Kluwer Health. A special appreciation regarding Professor Jun Wu and co-workers, from the magnificent “World of China”, for their impressive effort to establish a new miliary stone in the era of modern medicine with a new scientific “translational journal”. Congratulations to the members of the International Editorial Board. We suggest a motto to remark this exiting initiative: To look back for thinking ahead.

**Table 1:** 

Access this article online	
**Quick Response Code:**	**Website:** www.burnstrauma.com
	**DOI:** 10.4103/2321-3868.118922

We are grateful to everybody: Researchers, international scientists, clinicians, and academicians for their personal and successful contributions to promote a cultural challenge toward a new horizon in the field of translational research (TR). We enjoy to work together with the common objective to perform continuous medical education programs, exploring the methods in research, designing study, and to improve multidisciplinary and multiprofessional collaboration in the basic sciences and in the human trials. We have the common target to implement knowledge for a better understanding of physiological functions, the identification of the multitude of trigger points to realize the mechanisms causing illness and syndromes.

The method of study design and the selection of the patients’ enrollment represent the decisive elements to transfer the data and taking into account the multiple variables through the analysis of the process necessary to validate the results, to establish costs-effectiveness of the research, and the decision making for the prevention and the management procedures in the health system. Furthermore, the skills standardization and maximizing the quality of care require a strict reasoning for audit control of the experimental model and the population studies. These elements are the mirror of the translation science where the main factors for interprofessional and transdisciplinary interventions are expressed by the triad: Speedy recovery, good outcome, and the quality of life.

## Definition of foundations

The epistemology is a branch of philosophy that studies the nature of knowledge, its presuppositions and foundations, its extent and validity. Carson *et al.*,[1] defined this term as the relationship between the researcher and the reality or how this reality can be known. There is *a priori* knowledge, or knowledge that is automatically known apart from experience and *a posteriori* knowledge that is gained from experience.

Epistemology deals with the nature of knowledge instead of the how-to of knowledge. Defined narrowly, epistemology is the study of knowledge and justified belief. Epistemology is concerned with the following questions: What are the necessary and sufficient conditions of knowledge? What are its sources? What is its structure, and what are its limits?

As the study of justified belief, epistemology aims to answer some other questions:

1. Is justification internal or external to one’s own mind?
2. What makes justified beliefs justified?
3. How we are to understand the concept of justification?

More broadly, epistemology is about issues having to do with the creation and dissemination of knowledge in particular areas of inquiry as defined and revisiting at the beginning of the last decades. Ontology is the nature of reality;[1] according to the positivist ontology there is a single, external, and objective reality to any research question regardless of research by initially identifying the topic, constructing appropriate questions and hypotheses to adopt a suitable research methodology[2] They also attempt to remain detached from the participants of the studies by creating distance between themselves and the participants. Especially, this is an important step in remaining emotionally neutral to make clear distinctions between reason and feeling as well as between science and personal experience. Positivists also claim the importance to clearly distinguish between fact and value judgment.

## Translational research in medicine

There is growing evidence about the role of TR in the field of medicine for a good medical practice; even though the definitions and meaning of translational science, translational medicine (TM), and clinical medicine need to be further clarified. TR and clinical medicine represent a crowded and complex network comprehensive of scientific and regulatory investigations translating preclinical researches with a specific emphasis on the biomaterials, bioengineering, Nano and biotechnologies, disease-specific biomarkers, cellular and molecular medicine, regeneration medicine, omics science, bioinformatics, applied immunology, molecular imaging, drug discovery and development, regeneration pharmacology and regulation of health policy. It is believed that TR will benefit and improve novel diagnostics/prognostics and therapeutics options for clinical use, postgenomic knowledge and experience as new disciplines that reflect additional levels of complexity. We should clarify the bioethics at the interface and paradigms between technology and society, academy and industries, as well as publics and private models.

TM should meet the demands to maintain or expand the biomedical workforce and education programs that attract and retain young people in the translational and biomedical sciences. It represents a young and promising entity arised between the end of 2nd millennium. Since that: Is the way things became important to remark that the increased number of people have been studied and applied the practice of such topics from 1993, just 2 decades ago.

The definition of TR is less clear than the definitions of basic and clinical research. Medline search indicates that the term TR appeared recently with relatively few references about cancer[3,4] (e.g., immunology studies spanning basic and clinical research) or work spanning disciplines within a particular type of research (e.g., bench research involving molecular genetics and immunology.[5]

With this present contribution, we invite the members of the editorial board of Burns & Trauma journal to encourage submitting, in a special section, their personal experience about the philosophy of “Translation research”. If this has a chance, welcome to the researchers, clinicians, and the allied people for their decisive contributions to strengthen the importance of a common way (researchers and clinicians) about the principles and methods of basic and clinical research. For example, in medicine it is used to “translate” findings in basic research into medical practice and meaningful health outcomes. Applying knowledge from basic science and testing the evidence in the clinical practice represent an impressive and ongoing challenge. The term translation may seem like an automatic part of research and medical practice, but in the real life, it is a major stumbling block in science and in the public health. This is partly due to the compartmentalization within sciences and research training.[6]

TM represents a scientific research useful for practical applications that enhance human health and well-being. It is practiced and removed continuously in the field of medical sciences, as a result of environmental change and social requirements. Basic scientists are not generally trained to think of the clinical application of their work; clinicians are often not taught to formulate research studies based on clinical observations; and public health scientists may not have a strong and precise background. These groups may have in common a long partnership and collaboration, but as our knowledge grows and research becomes more complicated, it has mandatory that new ways of approaching health aspects are needed for seamless translation.

With its focus on multidisciplinary collaboration, TR has the potential to promote several goals and offering novel therapies for patients suffering illness, with multiple injuries, end-stage organ failure, or other clinical problems. TR and TM represent a dynamic entity making a link, a sort of bridge, “from bench to bedside,” or from laboratory experiments through clinical trials to point-of-care patient applications. TR includes two areas. One is the process of applying discoveries generated during research in the laboratory and in preclinical studies, to the development of studies in humans. The second area concerns research aimed at enhancing the adoption of best practices in the community. Cost-effectiveness on prevention and treatment strategies is also an important part of translational science.[7–9] According to this definition, TR is part of a unidirectional continuum in which research findings are moved from the researcher’s bench to the patient’s bedside and in the community.

The first stage of TR (T1) transfers knowledge from basic science to clinical research, while the second stage (T2) transfers findings from studies and clinical trials to practice settings, where the findings may evidence to improve health and outcome.

## Communications in science and messages from the Ivory Tower

Communication in science remained for long time an insuperable wall caused by intrinsic difficulty for common people to understand the mysterious world managed by a solitary researcher and scientist. But the more complex and powerful scientific findings and technological achievements got the more skepticism spread among the common people. Researchers and stakeholders tried to face this development by large-scale information and local and national campaigns.

Communication in science expanded world-wide in the Public Understanding of Science and Humanities (PUSH) phase meaning to establish consciousness of scientific research within the community. Since the web system quickly changed the way of information, science communication once more will adapt to new conditions, probably becoming very much more interactive to drive this process. Although, a minority of scientists have knowledge about the principles of science communication and even less than these are certainly up-to-date with communication advances. A large amount of dissemination activities of research findings is still based on a scientist’s monologue with detailed explanations nonintelligible to the media; the common people know little to nothing about how scientific advance work; so that in spite of their acquiring knowledge through the different available and medical progress formats, this view is generally biased and often have little in common with reality. Hence, though communication of science to the public strongly increased within the last decades, research still seems to be a mystery.

The term Ivory Tower originates in the Biblical song[10] in which Solomon is extolling the beauty of his beloved, and as later used from the 20th century to designate a world or atmosphere, where intellectuals engage in pursuits that are disconnected from the practical concerns of everyday life. An Ivory Tower may also be an entity of “reason, rationality, and rigid structures colonizes the world of lived experience,” as explained by Kirsten J. Broadfoot *et al.*,[11,12] when pointed out the importance that scholarly work is founded in dialogue approach of organizational and cultural communication where a group inside the community or individually became able to create an essence of exclusivity and superiority. Broadfoot *et al.*, explained clearly that “functions like an exclusive club whose membership is tightly controlled by what might be called a “dominant frame.” In an academic sense, this leads to an “overwhelming and disproportionate dominance” typical for the diversity, care, innovation, and the construction of creative, care-full, healthy and cultural sensitive organization which may be typical of the Western world. In these circumstances, imaging of the Ivory Tower can be dangerous in its inherent privatization of knowledge and intellect.

“Ivory Tower” is often used to indicate philosopher, scientists, or opinion leaders that are detached from the real life. The metaphor of science living in an Ivory Tower is, therefore, not only describing a world locked away, but also portrays the fact of different linguistic, social, and habitual properties compared to the outside. So even if academy and research institutions become more open; the scientists, on the one side, and the regulators, journalists, policymakers, and the group of pressure as well as the common citizen might face difficulties to communicate as they do not have familiarity on the core knowledge of scientific progress.

Connections from the lab to the public exist, via, for example, press relations offices, news on institute web sites, television, public debate, or newspapers, and so on. However, the critical point is: What is perceived by the community that can generate impact? Communication means to convey meaningful information to create shared understanding. If messages from environmental science are not suitable to produce public endorsement, we remain in the Ivory Tower despite all connections there might be. This demands easily intelligible communication and also for subsequent confirmation of the right understanding. As environmental sciences live through a concern about the environment as a whole, disconnection from our outside is more than just a typical academic characteristic.

## Collaborative partnership in research-perspectives and conclusion

Despite progress in interdisciplinary research, difficulties remain. Miller *et al.*,[13] report that scholars, educators, and practitioners need to critically rethink the ways in which interdisciplinary research and training are conducted. The authors define epistemological pluralism as an approach for conducting innovative, collaborative research, and study. Epistemological pluralism recognizes that, in any given research context, there may be several valuable ways of knowing, and that accommodating this plurality can lead to more successful integrated study.

Focus on epistemological pluralism can facilitate the reorganization of interdisciplinary research and avoid the build-up of significant, but insufficiently integrative, disciplinary-dominated research. The same authors highlight how interdisciplinary work is impeded when divergent epistemologies are not recognized and valued, and that by incorporating a pluralistic framework, these issues can be better explored, resulting in more integrated understanding.
